# Appendiceal spirochaetosis in children

**DOI:** 10.1186/1757-4749-5-40

**Published:** 2013-12-13

**Authors:** Laurens J Westerman, Marguerite EI Schipper, Herbert V Stel, Marc JM Bonten, Johannes G Kusters

**Affiliations:** 1 Department of Medical Microbiology, University Medical Centre Utrecht, PO-box 85500, 3508 GA, Utrecht, The Netherlands; 2 Department of Pathology, University Medical Centre Utrecht, PO-box 85500, 3508 GA, Utrecht, The Netherlands; 3 Department of Pathology, Tergooiziekenhuizen, PO-box 10016, 1201 DA, Hilversum, The Netherlands

**Keywords:** Appendicitis, Spirochaetes, *Brachyspira* species, Human intestinal spirochaetosis, Appendiceal spirochaetosis, Real-time PCR

## Abstract

**Background:**

Acute appendicitis is a surgical emergency in which the appendix is surgically removed to prevent peritonitis due to perforation of the appendix. Depending on age and gender, up to 17% of removed appendices do not show the histopathological changes pathognomonic for acute appendicitis and are called ‘pseudo-appendicitis’. Intestinal spirochaetes have been reported in up to 12.3% of these non-inflamed appendices obtained from adults. Although children carry the highest risk for acute appendicitis, not much is known on the prevalence of intestinal spirochaetes in children. The aim of this study was to determine whether there is an association between pseudo-appendicitis and appendiceal spirochaetosis in children.

**Methods:**

Archival appendix specimens from paediatric patients (less than 18 years old) were obtained from two Dutch hospitals (acute appendicitis, n = 63; pseudo-appendicitis, n = 55; control appendices, n = 33) and microscopically analysed by H&E staining and spirochaete-specific immunohistochemistry and *Brachyspira* species specific real-time PCR.

**Results:**

Five out of 142 appendices were found to be positive, all in male patients: one in the acute appendicitis group, two in the pseudo-appendicitis group and two in the control group.

**Conclusion:**

The results obtained do not provide evidence for a role of *Brachyspira* species infection in the aetiology of acute appendicitis in children.

## Background

Acute appendicitis is a clinical syndrome characterized by peri-umbilical pain and/or pain in the right lower abdominal quadrant, anorexia, fever, vomiting, and signs of generalized or localized peritoneal irritation (guarding or rebound tenderness). Definitive proof of acute appendicitis is the histopathological evaluation of the removed appendix: acute appendicitis is characterized by a massive invasion of neutrophils in the entire appendiceal wall, usually combined with focal or extensive ulceration and/or obliteration of the mucosa. The pathogenic mechanisms for this inflammatory process have remained unclear, despite numerous research-efforts on this subject. The term pseudo-appendicitis is used to describe clinical conditions mimicking acute appendicitis, where the removed appendix fails to show the characteristic histopathological changes defining ‘true acute appendicitis’.

Numerous infectious agents have been implied in the aetiology of acute appendicitis, such as common intestinal pathogens (*Salmonella* species, *Campylobacter* species, *Clostridium* species, various intestinal parasites) and intestinal spirochaetes [1-7]. The latter are anaerobic gram-negative bacteria and are occasionally found in the colon and appendix of humans with abdominal complaints where they cause a condition called human intestinal spirochaetosis (HIS) [8]. Human intestinal spirochaetosis was first described by Harland and Lee in 1967 and is characterized by the attachment of spirochaetes to the epithelium of the colonic mucosa (Additional file 1: Figure S1) [9]. In 1982, Hovind-Hougen et al. reported the first culture of a spirochaete isolated from a colon biopsy-sample, and named it *Brachyspira aalborgi*[10]. Our knowledge regarding both the prevalence and pathogenic potential of these putative human pathogens is scarce, as they can only be cultured under strict anaerobic conditions. Therefore, microscopic examination of biopsy samples is the gold standard for diagnosing intestinal spirochaetes in humans, but sensitivity may decrease in acute appendicitis where the mucosal structure is disrupted. PCR detection on formalin-fixed paraffin-embedded (FFPE) samples does not depend on intact mucosal structure and also allows species identification, as was demonstrated recently [11].

In veterinary medicine, intestinal spirochaetes are recognized as important pathogens in pigs (*B. hyodysenteriae* and *B. pilosicoli*) [12,13] and poultry (*B. intermedia* and *B. pilosicoli)*[14-16]. Only two species have ever been isolated from humans: *Brachyspira aalborgi* and *Brachyspira pilosicoli*[11,17-19], and there are reports of a third species tentatively named “*B. hominis”*[11,20-22]. However, this species has recently been shown to be a 16S-rDNA variant of *B. aalborgi*[23]. While *B. pilosicoli* has been isolated on numerous occasions, *B. aalborgi* is notoriously difficult to culture, with only a few successful descriptions of its isolation in the literature [10,21,24-26].

Results from two recent studies provided evidence for an association between *B. pilosicoli* and inflammatory changes in colon biopsy specimens and *B. pilosicoli*-induced pathological changes in cultured Caco-2 cells in vitro [11,27]. Yet, the pathogenicity of this bacterium for humans has not been unequivocally demonstrated.

The first observation of spirochaetes and appendicitis stems from 1911. Thiroloix and Durand described a 35 year-old woman with acute appendicitis with a spirochaete isolated from her blood [28]. Twenty years later, in 1930, Mazza examined 394 appendices using light-microscopy and identified spirochaetes in 9.6%. Unfortunately, he did not differentiate between acute appendicitis and pseudo-appendicitis [2]. Since then the role of spirochaetes in appendices removed for either histopathological acute appendicitis, pseudo-appendicitis and/or other reasons has been investigated and a prevalence of up to 12.3% has been observed in pseudo-appendicitis [2-7]. However, all evidence stems from samples obtained from adults or is not corrected for age, whereas children are at risk for acute appendicitis [29]. The aim of this paper was to investigate whether *Brachyspira* species are associated with pseudo-appendicitis. This was tested by investigating the presence of *Brachyspira* species in appendices of patients between 2 and 18 years of age with acute appendicitis, pseudo-appendicitis and without clinical symptoms of appendicitis obtained from the histopathological archives of two Dutch hospitals.

## Materials and methods

### Ethics statement

Patients, and/or their legal representatives, visiting a Dutch hospital are actively informed of the ‘opt-out’ system regarding research on archival patient material. Dutch law requires all studies using such materials to obtain an official approval by the local ethics committee. This study was approved by the Medical Ethics Committee of the University Medical Centre Utrecht, The Netherlands, under protocol number 11-198/C. Only material from patients that did not opt-out has been included in this study.

### Selection of archival appendix-specimens

Formalin-fixed paraffin-embedded (FFPE) appendix resection specimens collected between May 1988 and February 2011 from paediatric patients (between 2 and 18 years old) were selected retrospectively from stored collections in two Dutch hospitals (University Medical Centre Utrecht, Utrecht and Tergooiziekenhuizen, Hilversum). Three clinico-pathological groups were created: histopathologically proven acute appendicitis, pseudo-appendicitis and a control group (surgically removed for non-acute, non-inflammatory pathology). Per group the aim was to select 60 samples, equally distributed over three age groups (2 < 6, 6 < 12 and 12 < 18 years) and with equal gender distribution within the group. Samples were selected consecutively in time (starting from 2010 and going back to 1988) from the automated archive, based on the following criteria: clinical suspicion of acute appendicitis (both true appendicitis and pseudo-appendicitis) or appendices removed for other surgical reasons (non-acute, non-inflammatory pathology). Samples were excluded if the pathology report mentioned total fibrous obliteration of the appendiceal mucosa, obstruction due to tumours or other architectural disturbances, extensive mucosal ulceration and when not enough material remained to process the FFPE-sample as required.

### Sample processing

Of each FFPE-sample, three 4 μm sections were cut and mounted on glass-slides for histopathology, and two subsequent 20 μm sections were cut and placed in two separate 1.5 ml Eppendorf tubes (Eppendorf, Hamburg, Germany), then three additional 4 μm controls were cut and mounted on glass-slides. The knife was then discarded and the microtome cleaned thoroughly with 96% ethanol to prevent contamination of the next FFPE-block with DNA from the previous sample. The tubes were frozen at -80°C until DNA isolation.

### DNA extraction

Samples were processed by a semi-automatic deparaffinization protocol on a VERSANT® kPCR Sample Prep Machine according to the protocol supplied by the manufacturer (Siemens Healthcare Diagnostics, Breda, The Netherlands). Each DNA-isolation run included a negative control (HPLC-grade water). Each sample was centrifuged at 15,000 *g* for one minute, then 700 μl buffer consisting of 10 mmol/L tris(hydroxymethyl)aminomethane (TRIS), 0.1 mmol/l ethylenediaminetetraacetic acid (EDTA), 50 g/l sodium dodecyl sulphate (SDS), pH 8.0) was added to each sample [30]. Samples were then incubated at 80°C for 30 minutes in a shaking heat-block (1,400 rpm). Subsequently, samples were centrifuged at 15,000 *g* for 30 seconds and 100 μl proteinase K and 40 μl Phocine Herpes Virus (PhHV, internal control) were added [31]. Samples were then incubated at 65°C for 30 minutes in a shaking heat-block (750 rpm). Subsequently, all samples were centrifuged at 15,000 *g* for five minutes and 500 μl of the supernatant was transferred to 5 ml sterile polypropylene round bottom tubes (BD Biosciences, Breda, The Netherlands) and 100 μl of HPLC-grade water was added to the sample. One hundred μl DNA-eluate was extracted from 500 μl of the sample according to the manufacturers instructions.

If the internal controls exceeded a Ct-value of 36 for either PhHV (suggesting inhibition of PCR) or β-globulin (suggesting insufficient quality of DNA), samples were manually deparaffinised from the second tube. Samples were excluded from further analyses if the Ct-values for the internal controls exceed the cut-off values again. The manual DNA isolation protocol for purification was described previously [11]. Briefly, several wash-steps consisting of xylene, ethanol, and acetone remove the paraffin, followed by digestion with proteinase K and heat-inactivation of proteinase K. DNA was extracted using the Siemens Healthcare Diagnostics VERSANT® kPCR Sample Prep Machine as described above.

### PCR conditions and sequencing

PCR reactions were performed in a LightCycler 480 II PCR machine (Roche Diagnostics, Almere, The Netherlands) as previously described, without species-specific probes [32]. All primers, probes and PCR-conditions can be found in Table 1. Species identification was based on sequencing of the *Brachyspira* specific 16S-rDNA present in the positive samples as previously described [11]. Obtained sequences were compared with known sequences using BLAST and the Ribosomal Database Project and submitted to GenBank [33,34].

**Table 1:** Primer-sets, amplicon length, annealing temperature, amplification time and PCR-protocols

**Primer-set**	**Name**	**5′–––3′**	**Amplicon**
Bspp	B.spp FW	AACATggACTAATACCgCATATAC	147 bp
	B.spp probe	LC610-TgAgCCTgCggCCTATTAgCC-BBQ	
	B.spp REV	CTCAggTCggCTACCTATC	
β-globulin	β glob FW (GH20)	gAAgAgCCAAggACAggTAC	268 bp
	β glob probe	Cy5-TCTgCCgTTACTgCCCTgT-BBQ	
	β glob REV (PC04)	CAACTTCATCCACgTTCACC	
PhHV	PhHV FW	gggCgAATCACAgATTgAATC	89 bp
	PhHV probe	LC640-TTTTTATgTgTCCgCCACCATCTggATC-BBQ	
	PhHV REV	gCggTTCCAAACgTACCAA	
Step A	Step A FW	TggATAAgTTAgCggCgAACTg	212 bp
	Step A REV	TCAggTCggCTACCTATCg	
Step B	Step B FW	gAgCCTgCggCCTATTAgC	253 bp
	Step B REV	gCCgAggCTTACATTATCTACTgTC	
Step C	Step C FW	AgCgACATCgCgTgAgg	258 bp
	Step C REV	TCCATCATCCCCTACAATATCCAAg	

PCR conditions.

Primer concentration: 2,5 μM of both forward and reverse primers.

Buffer composition.

Probe PCR: LightCycler® 480 Probes Master (Version 09).

Step A-C PCR: LightCycler® 480 SYBR Green I Master (Version 12).

PCR volume.

Probe: 13 μl buffer and 2 μl DNA-eluate.

Step A-C: 15 μl buffer and 5 μl DNA-eluate.

PCR consumables (probe and Step A-C).

White 96-well plate (Roche Diagnostics, Almere, The Netherlands) sealed with transparent self-adhesive foil (Roche Diagnostics, Almere, The Netherlands).

PCR protocol.

Probe PCR: pre-incubation at 95°C for 10 minutes, denaturing at 95°C for 10 seconds, amplification at 60°C one minute, 45 cycles.

Step A-C PCR: pre-incubation at 95°C for 10 minutes, denaturing at 95°C for 10 seconds, 10 seconds annealing at 62°C, amplification at 72°C one minute, 45 cycles. Melting-curve analysis: 5 seconds at 95°C, one minute at 65°C, 2.2°C per second increase of temperature until 97°C with five acquisitions per degree Celsius.

### Histopathology

The first and last 4 μm slides were stained with standard haematoxylin and eosin (H&E). All H&E-slides from all appendices were revised by an experienced pathologist specialized in gastrointestinal pathology (MEIS). Mucosal remains were scored using five categories: totally absent; possibly some remains; some remains; relatively normal mucosal remains and normal mucosa. For *Brachyspira* detection all appendices were subjected to immunohistochemistry (*Borrelia burgdorferi* (Lyme) IgG antiserum, ILP 0301, ImmunoLogic, Duiven, The Netherlands) on the second 4 μm slide after the first H&E-slide. This staining is based on the immunological cross-reactivity of a universal spirochaete antigen and is used for routine diagnostic purposes in most Dutch Pathology Departments to confirm the presence of spirochaetes in histopathological samples. The specificity of this staining for *Brachyspira* species was demonstrated in a previous study [11]. A five-point scale was used to score the result of this spirochaete specific colouring (negative (−); possibly positive (±); focally positive (+); positive (++) and strongly positive (+++)). The diagnosis of HIS was made if a slide scored at least focally positive (+).

### Statistical analysis

Statistical analyses was performed using IBM® SPSS® Statistics Version 20 (release 20.0.0) for Macintosh OS X.

## Results

151 archival samples were obtained (63 of patients with acute appendicitis, 55 of patients with pseudo-appendicitis and 33 control patients). No statistical differences existed within the groups regarding age, sex and histopathological diagnosis. Inhibition of PCR (PhHV) or DNA quality (β-globulin) occurred in 83 samples. As per protocol, DNA was re-extracted from a second sample and this resolved the inhibition in 74 samples, leaving 142 samples for analysis (59 with acute appendicitis (27 females and 32 males), 50 with pseudo-appendicitis (21 females and 29 males) and 33 in the control group (18 females and 15 males)).

The presence of intestinal spirochaetes was not mentioned in any of the original pathology-reports. Based on revision of H&E-slides and immunohistochemistry four (2,8%) samples were considered positive; one (1.7%) in the acute appendicitis group, one (2.0%) in the pseudo-appendicitis group and two (6.1%) in the control group. These four samples were also detected by real-time PCR (Table 2). One sample in the pseudo-appendicitis group was positive by real-time PCR, but not by immunohistochemistry, increasing the prevalence in this group to 4.0% (n = 2).

**Table 2:** Immunohistochemistry and PCR result

**PCR result**	**Immunohistochemistry**				
	**−**	**+/−**	**+**	**++**	**+++**
Negative	135	2	0	0	0
Positive	1	0	1	2	1

Sequencing revealed the 16S-rDNA variant of *B. aalborgi* in two patients (one acute appendicitis and one pseudo-appendicitis) and *B. aalborgi* in three patients (one pseudo-appendicitis and two control patients). These sequences have been submitted to GenBank (accession numbers JX463020 through JX463024). There was no statistical correlation between the presence of *Brachyspira* species and histopathological outcome (p = 0.54, Chi-squared).

Only appendices of males were found to be positive for *Brachyspira* species (p = 0.041, Fisher’s Exact test).

## Discussion

To the best of our knowledge, this is the first study investigating the association between *Brachyspira* species and appendices removed for clinically acute appendicitis in children. In addition, we are the first to perform species identification in appendiceal spirochaetosis, using 16S-rDNA sequencing.

Three previous studies observed a higher prevalence of appendiceal spirochaetosis in pseudo-appendicitis [3,5,7], whereas one did not (Table 3) [6]. A major difference with these previous studies, which were all microscopy-based, is that we used both light microscopy, real-time PCR and sequencing of positive samples to obtain species determination allowing us to confirm the microscopy data and differentiate between the *Brachyspira* species present. The latter might be important, as there is recent evidence suggesting an inflammatory response to the presence of *Brachyspira pilosicoli*[11,27]. As we failed to identify any *B. pilosicoli* in the appendices, we conclude that *B. pilosicoli* is not associated with pseudo-appendicitis in Dutch children. Only *B. aalborgi* was identified in some patients, however, the prevalence was low and there was no association between their presence and pseudo-appendicitis. Although our study size is somewhat smaller than three of the previous studies into appendiceal spirochaetosis, we found a higher prevalence of *Brachyspira* species in the control group (6.1%) versus the pseudo-appendicitis group (4.0%), however this was not statistically significant. By increasing the sample size we would, at best, find a marginal role for *Brachyspira* in the aetiology of pseudo-appendicitis. Comparing our results with literature, our prevalence in all groups is within the range of those previously reported (Table 3).

**Table 3:** Prevalence of appendiceal spirochaetosis

**Article (year of publishing)**	**Total appendices**	**Acute appendicitis**	**Pseudo-appendicitis**	**Control group**	**Other diagnoses**
Mazza (1930) [2]	38/394 (9.6%)	Not specified	Not specified	Not specified	Not specified
Lee (1971) [3]	62/790 (7.8%)	7/160 (4.4%)	51/523 (9.8%)	4/107 (3.7%)	Not specified
Takeuchi (1974) [4]	8/388 (2.1%)	Not specified	Not specified	Not specified	Not specified
Henrik-Nielsen (1985) [5]	18/671 (2.7%)	3/414 (0.7%)	13/106 (12.3%)	2/107 (1.9%)	0/44 (0%)
Yang (1997) [6]	4/109 (3.7%)	1/72 (1.4%)	1/16 (6.3%)	1/9 (11.1%)	1/12 (8.3%)
Haleem (2003) [7]	2/598 (0.3%)	0/473 (0%)	2/97 (2.1%)	Not specified	0/28 (0%)
Westerman (this study)	5/142 (3.5%)	1/59 (1.7%)	2/50 (4.0%)	2/33 (6.1%)	Not applicable

While PCR is, in general, considered to be more sensitive and specific than classical microscopy- or culture-based diagnoses, we found only one additional case using PCR versus immunohistochemistry specifically targeted at all spirochaetes. The use of two highly specific, independent techniques reduces the chance of false-positives, which is an important difference with previous studies on appendiceal spirochaetosis, since these were all H&E-stain based. This difference in used techniques might be an explanation as to why we did not confirm the previously reported association between *Brachyspira* species and pseudo-appendicitis. Alternatively, local differences in the prevalence of *Brachyspira* species or the fact that we included only children, whereas previous studies consisted predominantly of adults, might explain the differences in prevalence between our study and those previously performed.

A potential limitation of this study is that only appendices were available for analysis, as it might be hypothesised that the presence of *Brachyspira pilosicoli* in the colon, but not necessarily in the appendix, might be associated with the clinical symptoms of acute appendicitis. While this could be a limitation of microscopy-based studies as they can only observe the end-on attachment of *Brachyspira* species to the appendiceal mucosa, it is known from avian, canine, porcine and murine infections with *B. pilosicoli* that the presence of this bacterium is not limited to the end-on attachment to the mucosa, but is also present in faecal samples, indicating that it moves freely through the colon content [35,36]. Thus, it can be expected that, as most appendix-specimens contain some faecal matter, the presence of *B. pilosicoli* would also have been detected by PCR. In fact, finding one additional positive sample by PCR versus microscopy supports this assumption.

Another potential limitation might be that sequencing of part of the 16S-rDNA gene does not fulfil all necessary requirements for definitive species identification. However, as was shown before, the obtained fragment is sufficient to differentiate the human *Brachyspira* species from each other [11].

Remarkably, only appendices from males were found positive for *Brachyspira* species (p = 0.041). This association has been reported before, but often more males than females were included in those studies, whereas this study aimed to include equal numbers of males and females. Thus far no clear biological explanation for this phenomenon exists.

## Conclusion

Based on the results of this study there is no association between appendicitis and pseudo-appendicitis in Dutch children and the presence of Brachyspira species in the appendix.

## Abbreviations

HIS: Human intestinal spirochaetosis; PCR: Polymerase chain reaction; FFPE: Formalin-fixed paraffin-embedded; DNA: Deoxyribonucleic acid; rDNA: Ribosomal deoxyribonucleic acid; PhHV: Phocine herpes virus; Ct: Cycle time.

## Competing interests

The authors declare that they have no competing interests.

## Authors’ contributions

LJW conceived and carried out experiments, LJW, MEIS, HVS, MJMB and JGK conceived experiments and analysed data. All authors were involved in drafting the paper and had final approval of the submitted version.

## Supplementary Material

Additional file 1: Figure S1 Human intestinal spirochaetosis. The spirochaetes are present as a ‘false brush border’ attached to the mucosa (arrow), leaving the goblet cells unaffected. Appendix specimen, haematoxylin and eosin stain, original magnification 630 times, bar equals 20 μm. Click here for file

## References

[B1] Lamps LW Infectious causes of appendicitis Infect Dis Clin North Am 2010 5 995 1018 ix-x 10.1016/j.idc.2010.07.012 20937462

[B2] Mazza S Espiroquetosis apendiculares [Appendiceal spirochaetosis] Prensa Med Argent 1930 5 464 468

[B3] Lee FD Kraszewski A Gordon J Howie JG McSeveney D Harland WA Intestinal spirochaetosis Gut 1971 5 126 133 10.1136/gut.12.2.126 5548558 PMC1411533

[B4] Takeuchi A Jervis HR Nakazawa H Robinson DM Spiral-shaped organisms on the surface colonic epithelium of the monkey and man Am J Clin Nutr 1974 5 1287 1296 4447095 10.1093/ajcn/27.11.1287

[B5] Henrik-Nielsen R Lundbeck FA Teglbjaerg PS Ginnerup P Hovind-Hougen K Intestinal spirochetosis of the vermiform appendix Gastroenterology 1985 5 971 977 3972236 10.1016/s0016-5085(85)80016-0

[B6] Yang M Lapham R Appendiceal spirochetosis South Med J 1997 5 30 32 10.1097/00007611-199701000-00006 9003819

[B7] Haleem A Al-Hindi H Al Husseini H Al Juboury M Appendiceal spirochetosis: a light and electron microscope study of two cases Ann Saudi Med 2003 5 216 219 16985325 10.5144/0256-4947.2003.216

[B8] Tsinganou E Gebbers JO Human intestinal spirochetosis–a review Ger Med Sci 2010 5 Doc01 20200654 10.3205/000090 PMC2830567

[B9] Harland WA Lee FD Intestinal spirochaetosis BMJ 1967 5 718 719 10.1136/bmj.3.5567.718 6038367 PMC1843001

[B10] Hovind-Hougen K Birch-Andersen A Henrik-Nielsen R Orholm M Pedersen JO Teglbjaerg PS Thaysen EH Intestinal spirochetosis: morphological characterization and cultivation of the spirochete *Brachyspira aalborgi* gen. nov., sp. nov J Clin Microbiol 1982 5 1127 1136 6186689 10.1128/jcm.16.6.1127-1136.1982 PMC272552

[B11] Westerman LJ Stel HV Schipper MEI Bakker LJ Neefjes–Borst AE van den Brande JHM Boel CHE Seldenrijk CA Siersema PD Bonten MJM Kusters JG Development of a real-time PCR for identification of *Brachyspira* species in human colonic biopsies PLoS One 2012 5 e52281 10.1371/journal.pone.0052281 23284968 PMC3527525

[B12] Harris DL Glock RD Swine dysentery. I. Inoculation of pigs with *Treponema hyodysenteriae* (new species) and reproduction of the disease Vet Med Small Anim Clin 1972 5 561 565 4480857

[B13] Glock RD Harris DL Swine dysentery. II. Characterization of lesions in pigs inoculated with *Treponema hyodysenteriae* in pure and mixed culture Vet Med Small Anim Clin 1972 5 65 68 4480858

[B14] McLaren AJ Trott DJ Swayne DE Oxberry SL Hampson DJ Genetic and phenotypic characterization of intestinal spirochetes colonizing chickens and allocation of known pathogenic isolates to three distinct genetic groups J Clin Microbiol 1997 5 412 417 9003607 10.1128/jcm.35.2.412-417.1997 PMC229591

[B15] Stephens CP Hampson DJ Intestinal spirochete infections of chickens: a review of disease associations, epidemiology and control Anim Health Res Rev 2001 5 83 91 11708751

[B16] Trott DJ Stanton TB Jensen NS Hampson DJ Phenotypic characteristics of *Serpulina pilosicoli* the agent of intestinal spirochaetosis FEMS Microbiol Lett 1996 5 209 214 10.1111/j.1574-6968.1996.tb08432.x 8810504

[B17] Mikosza AS Hampson DJ Human intestinal spirochetosis: *Brachyspira aalborgi* and/or *Brachyspira pilosicoli*? Anim Health Res Rev 2001 5 101 110 11708739

[B18] Sato H Nakamura S Habano W Wakabayashi G Adachi Y Human intestinal spirochaetosis in northern Japan J Med Microbiol 2010 5 791 796 10.1099/jmm.0.017376-0 20378723

[B19] Mikosza AS La T de Boer WB Hampson DJ Comparative prevalences of *Brachyspira aalborgi* and *Brachyspira (Serpulina) pilosicoli* as etiologic agents of histologically identified intestinal spirochetosis in Australia J Clin Microbiol 2001 5 347 350 10.1128/JCM.39.1.347-350.2001 11136797 PMC87728

[B20] Jensen TK Teglbjaerg PS Lindboe CF Boye M Demonstration of *Brachyspira aalborgi* lineages 2 and 3 in human colonic biopsies with intestinal spirochaetosis by specific fluorescent in situ hybridization J Med Microbiol 2004 5 341 343 10.1099/jmm.0.05402-0 15017292

[B21] Jensen TK Boye M Ahrens P Korsager B Teglbjaerg PS Lindboe CF Moller K Diagnostic examination of human intestinal spirochetosis by fluorescent in situ hybridization for *Brachyspira aalborgi*, *Brachyspira pilosicoli*, and other species of the genus *Brachyspira (Serpulina)* J Clin Microbiol 2001 5 4111 4118 10.1128/JCM.39.11.4111-4118.2001 11682538 PMC88495

[B22] Pettersson B Wang M Fellstrom C Uhlen M Molin G Jeppsson B Ahrne S Phylogenetic evidence for novel and genetically different intestinal spirochetes resembling *Brachyspira aalborgi* in the mucosa of the human colon as revealed by 16S rDNA analysis Syst Appl Microbiol 2000 5 355 363 10.1016/S0723-2020(00)80065-X 11108014

[B23] Westerman LJ Stel HV Schipper MEI Ahad DSA Bonten MJM Hampson DJ Wagenaar JA Kusters JG Molecular Characterization of Human Intestinal Spirochaetosis Sixth International Conference on Colonic Spirochaetal Infections in Animals and Humans 2013 University of Surrey, Guildford, United Kingdom: University of Surrey 17

[B24] Calderaro A Villanacci V Conter M Ragni P Piccolo G Zuelli C Bommezzadri S Guegan R Zambelli C Perandin F Rapid detection and identification of *Brachyspira aalborgi* from rectal biopsies and faeces of a patient Res Microbiol 2003 5 145 153 10.1016/S0923-2508(02)00014-1 12648729

[B25] Brooke CJ Riley TV Hampson DJ Evaluation of selective media for the isolation of *Brachyspira aalborgi* from human faeces J Med Microbiol 2003 5 509 513 10.1099/jmm.0.05105-0 12748271

[B26] Kraaz W Pettersson B Thunberg U Engstrand L Fellstrom C *Brachyspira aalborgi* infection diagnosed by culture and 16S ribosomal DNA sequencing using human colonic biopsy specimens J Clin Microbiol 2000 5 3555 3560 11015363 10.1128/jcm.38.10.3555-3560.2000 PMC87436

[B27] Naresh R Song Y Hampson DJ The intestinal spirochete *Brachyspira pilosicoli* attaches to cultured Caco-2 cells and induces pathological changes PLoS One 2009 5 e8352 10.1371/journal.pone.0008352 20020053 PMC2791440

[B28] Thiroloix J Durand A Spirochétémie au cours d’une appendicite aiguë [Spirochaetaemia during acute appendicitis] Bull Mem Soc Med Hop Paris 1911 5 653 662

[B29] Addiss DG Shaffer N Fowler BS Tauxe RV The epidemiology of appendicitis and appendectomy in the United States Am J Epidemiol 1990 5 910 925 2239906 10.1093/oxfordjournals.aje.a115734

[B30] Bohmann K Hennig G Rogel U Poremba C Mueller BM Fritz P Stoerkel S Schaefer KL RNA extraction from archival formalin-fixed paraffin-embedded tissue: a comparison of manual, semiautomated, and fully automated purification methods Clin Chem 2009 5 1719 1727 10.1373/clinchem.2008.122572 19617290

[B31] Niesters HG Clinical virology in real time J Clin Virol 2002 5 Suppl 3 S3 S12 12467772 10.1016/S1386-6532(02)00197-X PMC7128152

[B32] Westerman LJ de Boer RF Roelfsema JH Friesema IH Kortbeek LM Wagenaar JA Bonten MJ Kusters JG Brachyspira species and gastroenteritis in humans J Clin Microbiol 2013 5 2411 2413 10.1128/JCM.01069-13 23637299 PMC3697658

[B33] Zhang Z Schwartz S Wagner L Miller W A greedy algorithm for aligning DNA sequences J Comput Biol 2000 5 203 214 10.1089/10665270050081478 10890397

[B34] Cole JR Wang Q Cardenas E Fish J Chai B Farris RJ Kulam-Syed-Mohideen AS McGarrell DM Marsh T Garrity GM Tiedje JM The Ribosomal Database Project: improved alignments and new tools for rRNA analysis Nucleic Acids Res 2009 5 D141 D145 10.1093/nar/gkn879 19004872 PMC2686447

[B35] Muniappa N Duhamel GE Mathiesen MR Bargar TW Light microscopic and ultrastructural changes in the ceca of chicks inoculated with human and canine *Serpulina pilosicoli* Vet Pathol 1996 5 542 550 10.1177/030098589603300509 8885181

[B36] Sacco RE Trampel DW Wannemuehler MJ Experimental infection of C3H mice with avian, porcine, or human isolates of *Serpulina pilosicoli* Infect Immun 1997 5 5349 5353 9393839 10.1128/iai.65.12.5349-5353.1997 PMC175772

