# Supplementary figures and images for: Appendiceal spirochaetosis in children
Source: Gut Pathog. 2013 Dec 13;5:40. doi: 10.1186/1757-4749-5-40 (PMC4029455; doi:10.1186/1757-4749-5-40)

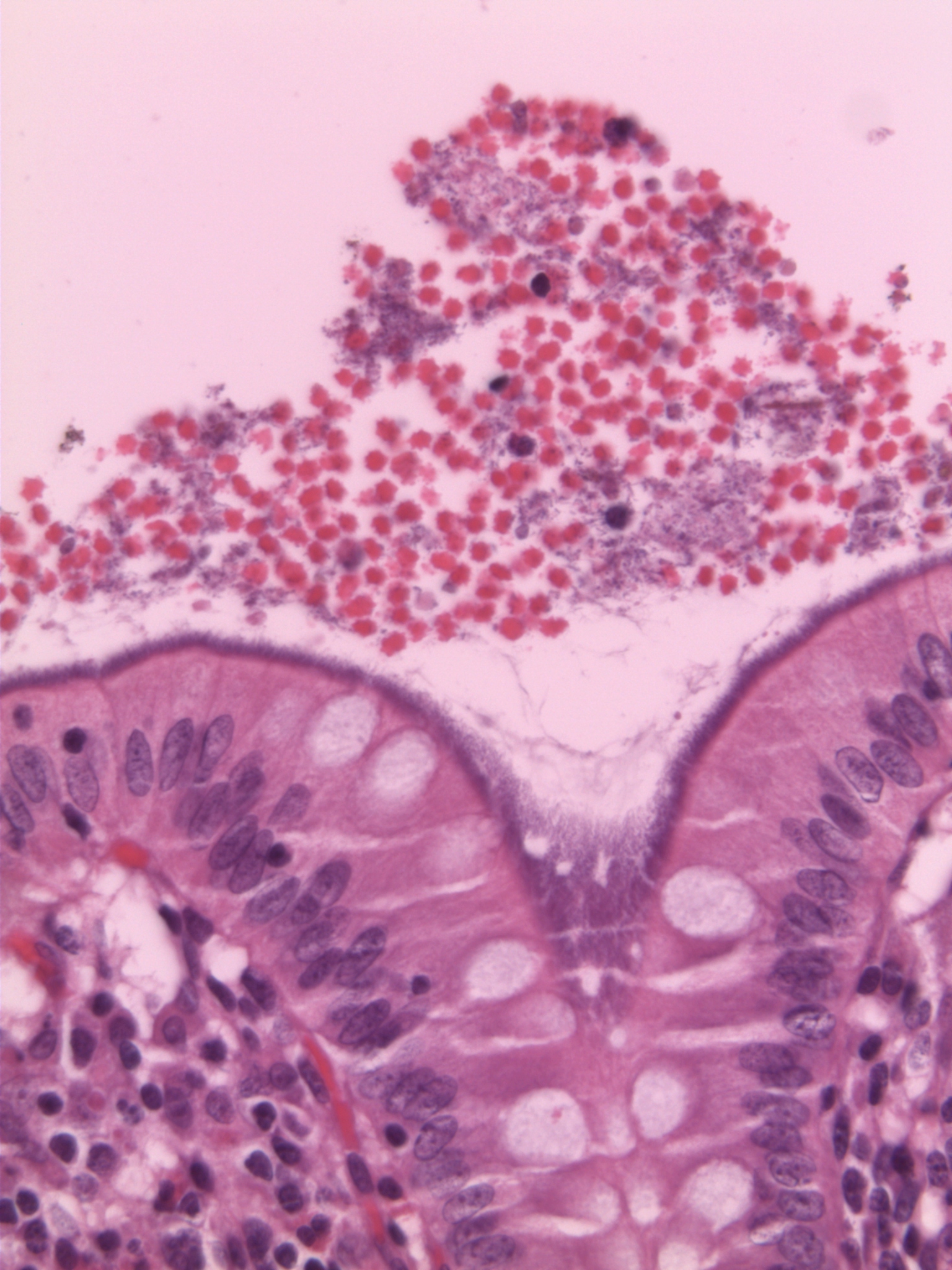

Supplement: Additional file 1: Figure S1 — Human intestinal spirochaetosis. The spirochaetes are present as a ‘false brush border’ attached to the mucosa (arrow), leaving the goblet cells unaffected. Appendix specimen, haematoxylin and eosin stain, original magnification 630 times, bar equals 20 μm. [file 1757-4749-5-40-S1.jpg]
